# The Effects of Standing Working Posture on Operation Force and Upper Limb Muscle Activation When Using Different Pointing Devices

**DOI:** 10.3390/ijerph191610217

**Published:** 2022-08-17

**Authors:** Jeewon Choi, Yu Lin, Ping Yeap Loh

**Affiliations:** 1 Department of Industrial and Management Systems Engineering, Dong-A University, 840, Hadan 2-dong, Saha-gu, Busan 604-714, Korea; 2 Graduate School of Design, Kyushu University, 4-9-1 Shiobaru, Minami-ku, Fukuoka 815-8540, Japan; 3 Department of Life Design and Science, Faculty of Design, Kyushu University, 4-9-1 Shiobaru, Minami-ku, Fukuoka 815-8540, Japan

**Keywords:** working posture, sitting, standing, pointing devices, muscle activation

## Abstract

This study investigated how sitting and standing working postures affected operation force, upper limb muscle activation, and task performance using different pointing devices. Fifteen male participants completed cursor aiming and dragging tasks using a conventional mouse, a vertical mouse, and a trackball at sitting and standing workstations. A custom-made force plate was used to measure operation forces applied to the pointing devices. Surface electromyography (EMG) was used to capture the activation of the biceps brachii, triceps brachii, deltoid, and trapezius. Task performance was measured by task success rates, and subjective ratings were obtained for the force required for operation, smoothness of operation, accuracy, and local fatigue in the upper limb. We quantified the following significant outcomes: (1) greater operation forces were found when standing; (2) standing reduced EMG amplitudes of the triceps and trapezius muscles for all tasks; (3) during the aiming task, the vertical mouse had greater operation forces; (4) during the dragging task, both the vertical mouse and trackball had greater operation forces; and (5) task success rates differed for pointing devices only when sitting. This study revealed the distinct biomechanical properties of standing working posture and suggested limited beneficial effects of alternative pointing devices in terms of task performance and subjective ratings.

## 1. Introduction

Emerging biomechanical evidence has suggested that excessive sedentary office work results in a higher risk of developing musculoskeletal disorders, such as lower back pain, rounded shoulders, and forward head [1,2,3]. Related reports focusing on perceived exertion during sedentary work have also indicated that most exertion occurs in the shoulder, neck, lower back, and arms [4,5]. Various interventions have been developed to reduce the time required for sedentary work, and the use of standing desks has been popularized in the recent literature [6,7,8]. Previous studies have demonstrated the effectiveness of standing workstations, which can reduce activation of the neck and shoulders muscles and lower back pain [9,10,11]. Furthermore, this was related to improved work comfort, task performance, and even academic outcomes compared to the conventional working posture of sitting [12,13]. Several vascular studies have also indicated its effectiveness in improving metabolic health and reducing the morbidity risk of heart disease [14,15]. Despite the benefit of standing desks, appropriate sitting time is still needed and should occur as well as standing time because excessive standing could possibly cause leg fatigue [16,17], while alternating sit-stand patterns effectively decrease activation of back muscles and discomfort [12,18].

Despite interventions to improve the health of office workers, the types of office work devices used, such as keyboards and mice, and the required motion for their operation, have not notably changed. Furthermore, among the many possible risk factors in the office environment, keyboard and mouse operations are suggested to influence occupational symptoms such as carpal tunnel syndrome (CTS) [19,20] and self-reports of shoulder and arm pain [21,22]. The duration of mouse operation is reported to account for approximately 63% of total computer working time, [23] and is known to be more strongly associated with an increased risk of CTS than keyboard use [24]. This is probably due to the rapid development of the graphic user interface, which allows many computer tasks to be performed through simple mouse movements rather than complicated keyboard input. Furthermore, mouse operations, such as moving, clicking, and dragging are widely preferred in many computer-based programs, such as games, because this facilitates immediate and precise input/reactions on computer screens [25].

Although the conventional type of mouse, which lets users place their hands on it with approximately 90-degree pronation of the forearm, has been used for decades, reports of mouse-related musculoskeletal symptoms have increased, mainly due to excessive wrist, upper-arm, and shoulder movements in the horizontal and frontal planes, as well as unnatural posture of the forearm during its use [26,27]. Specifically, prolonged compression of the median nerve of the forearm is a primary risk factor for CTS [28,29,30]. To address this issue, alternative pointing devices such as vertical mice and trackballs were introduced. The vertical mouse is designed to decrease the pronation angle and share the operation load on the wrist with the fingers. This mechanical structure is believed to potentially reduce the load on the wrist and median nerve, although its practical benefits remain controversial [31,32]. One study pointed out that even 16 h of familiarization sessions with the vertical mouse were insufficient for acclimation [33]. On the other hand, the trackball is operated by rotating a sphere placed on the top with only one or two fingers, which could reduce frequent wrist and shoulder movements. Several studies have indicated that the trackball could decrease activation of the shoulder muscles and angles of wrist flexion and shoulder abduction, compared to a conventional mouse [34,35].

The literature on biomechanics and occupational health contains reports on different types of alternative pointing devices involving the measurement of upper limb muscle activation and postural change [32,36,37]; nonetheless, there has been no single approach to define how different workstations with varied sitting and standing postures affect task performance and biomechanical variables, when alternative pointing devices are used. Therefore, the current study measured not only upper limb electromyography (EMG) patterns of the biceps brachii (BB), triceps brachii (TB), deltoid (DE), and trapezius (TR) muscles, which are related to the movements when controlling pointing devices, but also the operation force, particularly the vertical force applied to pointing devices. The operation force applied is an effective indicator for evaluating and identifying device control performance and the physical characteristics of device control strategies in real time [38]. Previous studies have measured the operation force to examine the relationships among task difficulty, working hours, the physical load in mouse work [39,40], and control strategies for the touchpad [41].

In this study, we investigated how operation force, upper limb muscle activation, task performance, and subjective ratings when operating three different pointing devices (conventional mouse, vertical mouse, and trackball) changed for different working postures with the interventions of sitting and standing workstations. The participants performed aiming and dragging tasks using these pointing devices during the experimental session and, at the same time, the task success rate was calculated. This study used a custom-made force plate to continuously measure the ground reaction force generated by arm and wrist movements using pointing devices. A primary hypothesis was that a standing posture would change the patterns of operation force and muscle activation when using different pointing devices compared with a sitting posture. We also hypothesized that alternative pointing devices would be beneficial in terms of task performance and subjective ratings.

## 2. Materials and Methods

### 2.1. Participants

Convenience sampling was used to recruit participants in this study. Fifteen right-handed healthy young participants (age: 24.9 ± 2.5 yr, height: 172.5 ± 5.2 cm, weight: 62.7 ± 7.5 kg), without known upper limb musculoskeletal disorders, were recruited. The handedness of participants was determined using the Edinburgh Handedness Inventory [42]. Prior to the experiment, all participants provided informed consent, in accordance with the approval of the Research Ethics Committee of the Faculty of Design, Kyushu University.

### 2.2. Experimental Setup

**Figure 1:**
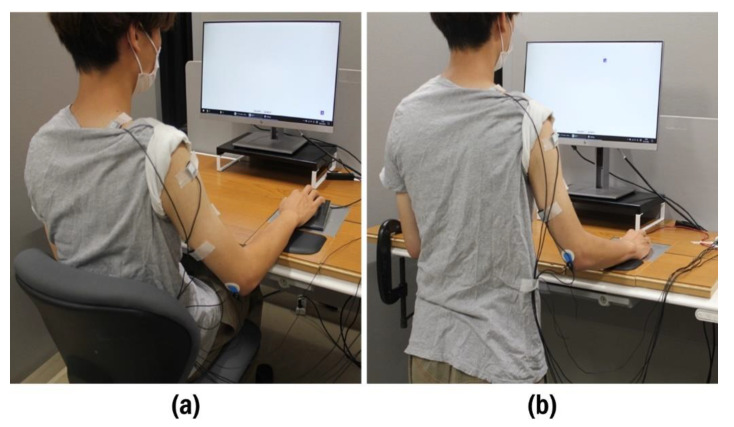
Experimental setups of (**a**) sitting and (**b**) standing workstations.

Anthropometric measurements were performed on each participant in a seated and standing position using standard procedures. Each participant was then seated on a chair in front of a height-adjustable desk (3S20LR-MY31; Okamura Corporation, Tokyo, Japan) and looked at a 25-inch monitor (resolution: 1920 × 1280 pixels, refresh rate: 60 Hz), which was placed on the desk approximately 0.5 m away from the participant at eye level. The chair height was adjusted to allow 90° knee flexion, with the feet resting on the floor. The desk height was also adjusted such that it was aligned with the participant’s seated elbow height (Figure 1a). The participant was then asked to stand up in front of the desk, and the desk height was adjusted such that it was aligned with the participant’s standing elbow height (Figure 1b). The preferred table heights for seated and standing work were 71.6 ± 3.1 and 104.8 ± 5.9 cm, respectively.

**Figure 2:**
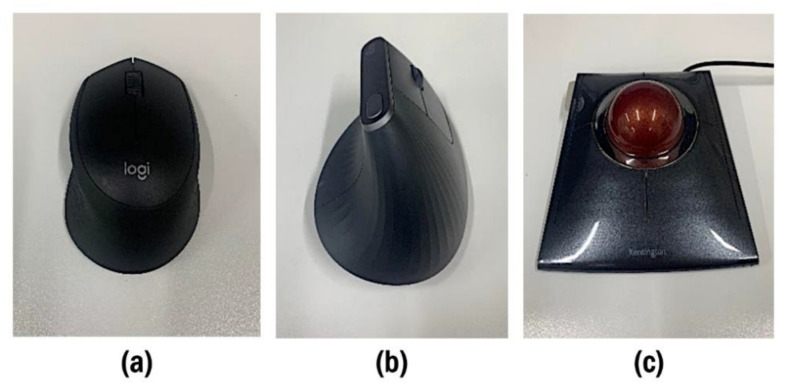
Pointing devices used in this study; (**a**) conventional mouse, (**b**) vertical mouse, and (**c**) trackball.

**Figure 3:**
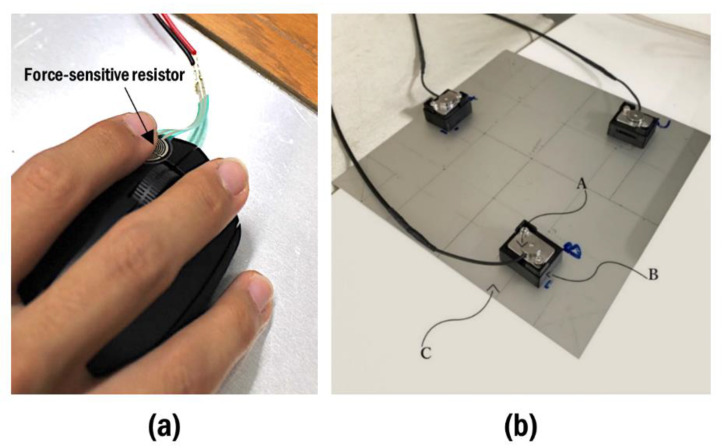
Force sensors used in the experiment. (**a**) A force-sensitive resistor secured on the left button of pointing device; (**b**) the internal structure of the custom-made force plate used in the experiment; (**A**) load cell, (**B**) loading fixture, and (**C**) the base of the force plate.

The top of the desk was covered with a plywood board (thickness:2 cm); on the right side of the desk, a pointing device was placed according to the conditions and used by the participants throughout the trials. This study involved three different pointing devices: a conventional mouse (M280B, Logicool, Tokyo, Japan) (Figure 2a), a vertical mouse (MX Vertical ergonomic mouse, Logitech) (Figure 2b), and a trackball (SlimBlade Trackball 72327JP, Kensington, Redwood City, CA, USA) (Figure 2c). A force-sensitive resistor (FSR-400; Interlink Electronics Inc., Camarillo, CA, USA) was secured on the left button of the pointing device to record activation timing offline. Under the range of pointing device movement, a force plate, with three loadcells (MCSR-5L-FG, Toyo Sokki Co., Ltd., Kanagawa, Japan), arranged into a triangular layout secured to the bottom of an aluminum plate (200 mm × 200 mm × 5 mm), was mounted flush with the plywood board (Figure 3).

### 2.3. Aiming and Dragging Task

This study used Compass 3.0 (Koester Performance Research, Ann Arbor, MI, USA), which is specialized software for measuring and analyzing users’ skills required for interacting with a computer. The representation of the pointing device was shown on the monitor screen as a conventional arrow-shaped white cursor for all conditions. Two types of tasks were included in this study: aiming (move-and-click) and dragging (drag-and-drop). For the aiming task, a series of targets (blue square; 48 × 48 pixels) appeared on the screen, one at a time. Participants were asked to aim at the target by moving the cursor and clicking as quickly as possible. If it was correctly aimed, the target disappeared and the next target appeared concurrently at a random location. For the dragging task, a target object (conventional yellow folder icon; 48 × 48 pixels) and a destination (grey trash bin icon; 48 × 48 pixels) appeared on the screen. Similar to the aiming task, when the target was correctly dragged into the destination, the pair disappeared, and the next target-destination pair appeared concurrently at a random location. For both tasks, 1 s of pause was given between trials. The distances between the cursor and target (aiming task) and target and destination (dragging task) were randomly set to be either middle (approximately one-fifth the screen width apart) or long (approximately half the screen width apart). The participant was asked to finish each trial within 10 s and was given no feedback on whether the trial was successful.

### 2.4. Procedure

Each participant took part in one experimental session. Each session began with anthropometric measurements and height adjustment of the chair and desk. Once the workstation was optimally configured, the participant was trained for the aiming and dragging tasks and familiarized with the experimental setup. Following the practice phase, the participants completed six experimental blocks for combinations of three pointing devices and two desk setups, each of which included 100 trials for each task. The order of the combinations and experimental tasks was systematically varied across blocks. Thus, one participant completed two tasks (aiming and dragging tasks) × 100 (trials) × three pointing devices (conventional mouse, vertical mouse, and trackball) × two workstations (seated and standing) = 1200 trials during the main phase. A short break of 5 min was provided between blocks, and the total session lasted approximately 2 h. The sequence of tasks, pointing devices, and workstations was randomized for all participants.

### 2.5. Data Acquisition

#### 2.5.1. Kinetics and Electromyography

The signals of the kinetic outputs measured from the three load cells of the force plate were amplified using a load cell amplifier (LA-1041, Toyo Sokki Co., Ltd., Kanagawa, Japan) and low-pass filter at 20 Hz. The force plate was set to zero prior to the test to cancel out the participant’s weight (the weight of the right hand). The signals were then digitized at a sampling rate of 200 Hz using an A/D converter (PowerLab 16/30; ADInstruments, Dunedin, New Zealand) before being stored on a computer.

Surface EMG was also recorded to determine the level of muscle activation in the four related muscles of the dominant arm (right) of the experimental task: the BB, TB, DE, and TR muscles. The surfaces of these muscles were palpated and cleaned with alcohol, and active EMG electrodes (BA-U410m; Nihon Santeku, Osaka, Japan) were placed on each surface along the direction of the muscle fiber in accordance with the SENIAM recommendations [43]. The ground electrode was attached to the right acromion and the head of the radius. EMG signals were amplified using a bio-instrumentation amplifier (BA1104m; Nihon Santeku) and band-pass filter (25–500 Hz). The EMG signals were digitized at a sampling rate of 1 kHz using the A/D converter and were transmitted to a computer.

Maximal voluntary contractions (MVCs) were measured for all tested muscles, which were used as a reference for normalizing the EMG signals. During MVC measurement, the participants performed at least three maximal contractions for each muscle. One MVC trial lasted for 5 s, and rests of 60 s were interspersed between trials.

#### 2.5.2. Task Performance

The results of each experimental aiming and dragging task in one trial block were aggregated after the measurement, and the outputs of the force-sensitive resistor and force plate were checked to identify valid click trials. Based on the validation, we randomly obtained 50 successful trials out of 100 valid trials. The task success rate was measured as the percentage of successful trials.

#### 2.5.3. Subjective Ratings

After one experimental block was completed, the participants rated their perceived comfort in using the pointing device. The questionnaire, which consisted of six questions, was formulated based on the ISO 9241-part 9 standard. This assessed the perceived levels of (1) force required for operation, (2) smoothness of operation, (3) accuracy, and fatigue of (4) finger, (5) wrist, and (6) arm. The scale ranged from one to five, with five representing the highest level.

### 2.6. Data Analysis

The digitized data were analyzed offline using LabChart 7 (ADInstruments) and Microsoft Excel. The operation forces (N) were calculated as the average of signals from the recordings of the three load cells. The criterion was defined as the duration between the onset and end of the cursor movement. The EMG signals of each tested muscle measured during the experimental tasks were full-wave rectified and normalized by the average rectified amplitude over the middle 3 s of the MVC trials (%MVC).

### 2.7. Statistical Analysis

A two-way analysis of variance (ANOVA) with repeated measures was applied to compare the effects of working posture at different workstations (sitting and standing) and pointing devices (conventional mouse, vertical mouse, and trackball) after checking for normality using the Shapiro–Wilk test. If the assumption of data normality was violated, analyses were performed using non-parametric Wilcoxon and Friedman tests. Bonferroni-corrected pairwise comparison was conducted as a post hoc test when a significant effect was observed. The statistical significance of the experimental conditions was determined as α = 0.05. Partial eta squared ($\eta_{p}^{2}$) was reported as a measure of effect size for significant results. All statistical analyses were conducted using SPSS Statistics (version 25.0; IBM, Armonk, NC, USA). For the biomechanical variables of operation forces and EMG amplitudes, values are reported as mean ± SD, while values of subjective ratings are reported as mean ± SE.

## 3. Results

### 3.1. Operation Forces and Electromyography Responses

**Figure 4:**
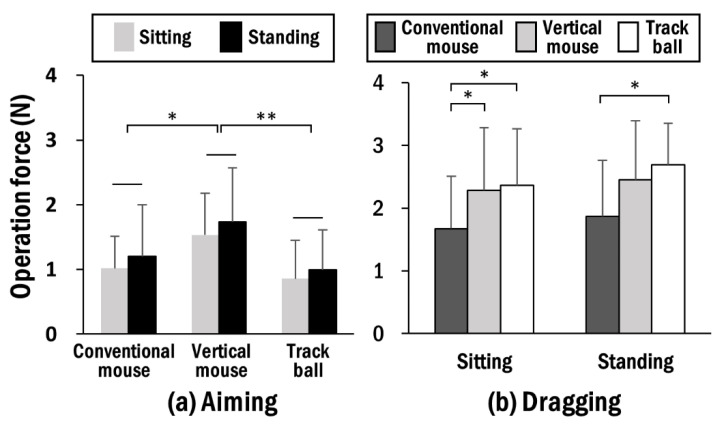
Average operation force (**a**) during aiming tasks by pointing device for working posture and (**b**) during dragging tasks by working posture for pointing devices. Asterisks denote a significant difference between pointing devices (* *p* < 0.05; ** *p* < 0.01).

The ANOVA tables are presented in Appendix A. During the aiming task, there was a significant main effect of working posture on the operation force. Specifically, greater operation forces were found in the standing posture (1.31 ± 0.80) than in the sitting posture (1.14 ± 0.68). The type of pointing device also had a significant effect on the operation force, with results of the corresponding post hoc tests indicating that the vertical mouse involved greater operation forces (1.63 ± 0.74) compared to the conventional mouse (1.11 ± 0.65) and the trackball (0.93 ± 0.60) (Figure 4a). No significant two-way interaction was observed.

The EMG amplitudes of the BB muscle were affected by the type of pointing device used, with the post hoc tests indicating that the vertical mouse had greater BB EMG amplitudes (0.75 ± 0.58) than the conventional mouse (0.59 ± 0.40). The main effects of working posture and the two-way interaction were not found to be significant. For the EMG amplitudes of the TB muscle, only working posture had a significant effect. Specifically, standing posture had lower TB EMG amplitudes (1.50 ± 0.82) than sitting posture (1.97 ± 1.10). For the EMG amplitudes of the DE and TR muscles, hardly any effects were found to be significant (Table 1).

During the dragging task, working posture had a marginal effect on the operation force, with standing posture involving a greater operation force (2.34 ± 0.93) than sitting posture (2.11 ± 0.91). Furthermore, the pointing device significantly affected the operating force. The post hoc tests indicated greater operation forces in use of the vertical mouse (2.37 ± 0.91) and trackball (2.53 ± 0.80) than in the conventional mouse (1.77 ± 0.91) (Figure 4b). There was no statistically significant two-way interaction observed.

While the EMG amplitudes of the BB and TB muscles were not affected by any factors or a two-way interaction, the pointing device had a significant effect on the DE EMG amplitudes, with the corresponding post hoc tests indicating higher values for the trackball (2.69 ± 1.94) than for the vertical mouse (2.09 ± 1.64). For the EMG amplitudes of the TR muscle, working posture had a significant effect. The post hoc tests showed that standing posture had lower TR EMG amplitudes (3.51 ± 3.00) than their counterpart (5.66 ± 4.32). The other variables did not show significant effects (Table 1).

### 3.2. Task Success Rate and Subjective Ratings

**Figure 5:**
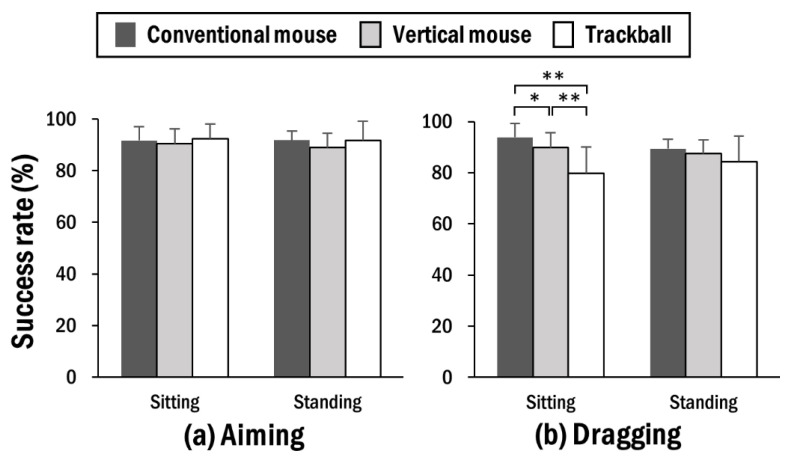
Average success rate during (**a**) aiming and (**b**) dragging tasks by working posture for pointing devices. Asterisks denote significant difference between pointing devices (* *p* < 0.05; ** *p* < 0.01).

During the aiming task, the task success rate was not influenced by working posture or pointing device used. The average task success rate was comparable between sitting (91.52 ± 5.13) and standing postures (90.88 ± 6.50) (Figure 5a). However, during the dragging task, the effects of the pointing device and two-way interaction were significant, with the post hoc tests indicating a significantly lower task success rate for trackball (82.03 ± 10.15) compared to the conventional mouse (91.59 ± 12.86). The post hoc tests for the interaction also demonstrated that sitting posture was associated with significantly lower values for use of trackball (79.69 ± 10.34), followed by vertical mouse (89.81 ± 6.26), and conventional mouse (93.88 ± 3.72) (Figure 5b).

**Figure 6:**
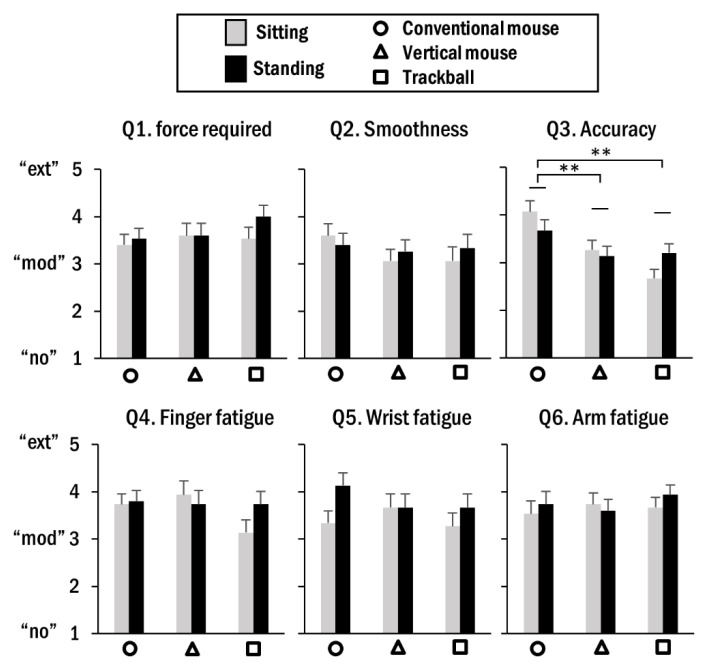
Average subjective ratings for force required for operation, smoothness of operation, accuracy, fatigue of finger, wrist, and arm. The labels on the vertical axis denote the level of each measure. The error bar indicates the SE and asterisks indicate significant difference between pointing devices (** *p* < 0.01).

For the six dimensions of subjective ratings measured during the aiming task, none of the terms were statistically significant. For the dragging task, non-parametric analyses revealed that only the perceived level of accuracy was significantly affected by the pointing device used ($\chi^{2}$(2) = 19.75, *p* < 0.01). Specifically, the conventional mouse was associated with a higher value (3.78 ± 0.17) than the vertical mouse (3.19 ± 0.13) and trackball (2.93 ± 0.13) (Figure 6).

## 4. Discussion

In this study, we compared the differences between sitting and standing postures in terms of biomechanical variables and subjective ratings when using conventional and alternative pointing devices. As hypothesized, the standing posture was associated with greater operational forces than the sitting posture, although the difference was marginal for the dragging task. The EMG results indicated lower amplitudes for the TB and TR muscles in the standing posture during the aiming and dragging tasks, respectively. During the aiming task, only the vertical mouse was associated with greater operating forces than the others, as well as with greater BB EMG amplitudes. During the dragging task, the standing posture did not affect the task success rate for all pointing devices, while the task success rate was reduced for the vertical mouse and trackball in the sitting posture. Furthermore, the results of the dragging task indicated that use of the vertical mouse and trackball were associated with greater operating forces than the conventional mouse.

One of the major findings of this study was that the operating forces on the pointing devices increased at the standing workstation. Previous work that considered touchscreen evidence showed that a standing posture caused a greater pushing force (up to 40%) compared to a sitting posture [44]. In terms of the inertia of the upper extremities, the aiming and dragging tasks would have similar effects to pushing in the touchscreen environment. The greater operation forces, however, were not associated with increased subjective ratings, such as the perceived level of force required. In [38], the average value of the maximum forces applied on the mouse button during MVC measurement was found to be as high as 53 N and the force applied on the button to range between 0.30 and 0.44 N. The average difference in the operation forces between the sitting and standing postures demonstrated in this study was similar to those observed in [38], ranging from 0.17 to 0.23 N for each task. We assumed that the participant could not differentiate between such small force changes. Importantly, although the subjective rating results regarding fatigue did not show a change between working postures, the authors suggest that the increased operation force in the standing posture could be a potential risk factor for musculoskeletal disorders in the long term [33,36].

The results obtained from the aiming task also indicated that the vertical mouse was used with greater operation forces than the conventional mouse and trackball. While the other pointing devices can be used with the forearm at approximately 90-degree pronation, the vertical mouse reduces the wrist pronation angle so that the primary wrist movement for mouse operation is changed from wrist flexion to ulnar deviation. Because greater maximal grip strength can be generated in ulnar deviation compared to wrist flexion, regardless of forearm position [45], the different structure of the vertical mouse might have contributed to increased operation forces. However, the greater operation force for using a vertical mouse should not lead to enhanced usability or reduced musculoskeletal risk. The results of the task success rate among different pointing devices during aiming and dragging tasks indicate that a greater operation force probably did not contribute to enhanced task performance. Furthermore, one study showed that the vertical mouse differs in wrist position, while pressure on the carpal tunnel remains unaffected [31]. On the other hand, although the two-way interaction was not significant, operation forces were the greatest when the vertical mouse was used in a standing posture during the aiming task. Considering the reduced base of support on the vertical mouse, securing an appropriate area of support on the working plane could be particularly important in maintaining postural stability in the standing posture [12]. This is because the working plane at the standing workstation is the only area that shares body weight, other than the base of support of the feet. Therefore, we assumed that participants could have exerted more operational forces on the vertical mouse to compensate for the decreased base of support.

Even if forearm movements are generally involved in the motions of operating pointing devices, the EMG amplitudes of the BB and TB muscles were generally less than 3% of MVC regardless of the working posture, as shown in previous studies [9,10]. On the other hand, we found that the EMG amplitudes of the TR muscle were relatively lower in the standing posture regardless of the experimental task and pointing device, while the DE EMG amplitudes were not affected. This partially confirms previous findings, where the standing posture had lower DE and TR activation than the sitting posture [9]. What makes these two experiments different is the trial (working) duration, which was set to 2 to 4 min in this study, while the previous trial duration studied was 45 min. Based on our results, we cautiously speculate that the altered DE activation observed in the previous study was likely the result of muscle co-contraction caused by musculature fatigue over a long trial period [46,47]. A possible explanation for the lower TR activation in the standing posture is that the participants tended to put more load on their forearms and wrists to support their body weight together with their feet. Hence, TR activation for shoulder abduction and postural maintenance of the upper limb can be reduced at a standing workstation.

The task success rate was not affected by any factor during the aiming task; however, it was greatly influenced by use of different pointing devices in the sitting posture during the dragging task. It is likely that the dragging task is relatively intense and requires more operational force compared to the aiming task, so that the difference in task performance is distinctly observed. The task success rates of the vertical mouse and trackball were lower than those of the conventional mouse in the sitting posture, and these patterns were duplicated in the results for perceived task accuracy. Because participants were long accustomed to using a conventional mouse with a high proficiency level at the sitting workstation, this might have caused them to perceive more difficulties in precisely controlling alternative pointing devices. However, such an effect would become less prominent at the standing workstation because cognitive load and attention resources could be moved from the external issues of operating pointing devices to maintaining internal postural stability with distinct kinetic strategies [48].

This study had several limitations. First, the effects of shape and size of the pointing devices were not considered in this study, and the experimental sessions were performed only on young male participants. These factors could limit the generalizability of the current findings to many pointing devices available in the market and their users, such as females and the elderly. On the other hand, although we measured the EMG amplitudes of the upper limb muscles, these muscles could not sufficiently cover the motions used for operating pointing devices in different working postures. Therefore, when investigating precise device operation and related fatigue, future studies should measure more closely the activation of forearm muscles, such as the flexor digitorum superficialis and flexor carpi radialis muscles.

## 5. Conclusions

From a biomechanical perspective, our findings have enhanced understanding of sit-stand interventions and suggested limited benefits of alternative pointing devices, the vertical mouse and trackball. Specifically, the standing posture involved significantly greater operation forces and the alternative pointing devices had distinct effects on the operation forces for standing and sitting postures. The beneficial effects of alternative pointing devices were not significant in terms of task performance and subjective ratings. Our results have important biomechanical implications regarding the pointing devices that could be effective in different workstations.

## Figures and Tables

**Table 1:** EMG amplitude results of (a) BB, (b) TB, (c) DE, and (d) TR muscles for each pointing device by working posture.

Pointing Devices	Conventional Mouse		Vertical Mouse		Trackball	
Postures	Sitting	Standing	Sitting	Standing	Sitting	Standing
*Aiming task*						
(a) BB EMG amplitude (%MVC)	0.61 ± 0.41	0.56 ± 0.40	0.82 ± 0.66	0.67 ± 0.49	0.60 ± 0.47	0.56 ± 0.45
(b) TB EMG amplitude (%MVC)	2.10 ± 1.26	1.59 ± 1.04	1.82 ± 1.10	1.48 ± 0.83	1.98 ± 0.95 ^a^	1.43 ± 0.57
(c) DE EMG amplitude (%MVC)	2.13 ±1.39	2.36 ± 1.85	1.86 ± 1.61	1.89 ± 1.39	1.74 ± 1.37	1.91 ± 1.31
(d) TR EMG amplitude (%MVC)	5.68 ± 4.17	4.10 ±3.26	4.95 ± 4.75	3.29 ± 2.83	5.65 ± 3.04	4.54 ±4.30
*Dragging task*						
(a) BB EMG amplitude (%MVC)	0.62 ± 0.44	0.60 ± 0.44	0.78 ± 0.70	0.65 ± 0.43	0.64 ± 0.51	0.61 ± 0.48
(b) TB EMG amplitude (%MVC)	2.22 ± 1.20	1.88 ± 1.46	2.37 ± 1.37	1.94 ± 1.49	2.30 ± 1.06	1.86 ± 0.52
(c) DE EMG amplitude (%MVC)	2.07 ± 1.79	2.39 ± 2.38	2.24 ± 1.42	1.93 ± 1.88	2.72 ± 1.91	2.67 ± 2.03
(d) TR EMG amplitude (%MVC)	6.53 ± 4.27 ^a^	3.75 ± 3.34	5.56 ± 5.41 ^a^	2.92 ± 2.13	4.89 ± 3.12	3.86 ± 3.54

^a^ *p* < 0.05; for significant difference compared with standing posture.

## Data Availability

The data that support the findings of this study are available from the corresponding author upon reasonable request.

## Appendix A. ANOVA Tables for Each of the Dependent Variables

**Sources**	**df**	**F**	***p*-Value**	** $\eta_{p}^{2}$ **
*Aiming task*				
*Operation force*				
Working posture	1	5.78	**0.03**	0.03
Pointing device	2	8.95	**0.00**	0.32
Interaction	2	0.01	0.91	0.00
*BB EMG amplitude*				
Working posture	1	2.03	0.18	0.04
Pointing device	2	5.21	**0.01**	0.13
Interaction	2	0.93	0.41	0.01
*TB EMG amplitude*				
Working posture	1	9.30	**0.01**	0.16
Pointing device	2	1.04	0.37	0.02
Interaction	2	0.27	0.77	0.01
*TR EMG amplitude*				
Working posture	1	3.32	0.09	0.07
Pointing device	2	0.97	0.39	0.02
Interaction	2	0.11	0.90	0.00
*DE EMG amplitude*				
Working posture	1	0.44	0.52	0.01
Pointing device	2	1.08	0.35	0.04
Interaction	2	0.18	0.84	0.00
*Task success rate*				
Working posture	1	0.37	0.55	0.01
Pointing device	2	2.00	0.15	0.06
Interaction	2	0.47	0.51	0.01
*Dragging task*				
*Operation force*				
Working posture	1	4.32	0.06	0.03
Pointing device	2	6.69	**0.00**	0.29
Interaction	2	0.23	0.79	0.00
*BB EMG amplitude*				
Working posture	1	2.98	0.11	0.03
Pointing device	2	2.24	0.13	0.07
Interaction	2	1.36	0.27	0.03
*TB EMG amplitude*				
Working posture	1	3.88	0.07	0.07
Pointing device	2	0.14	0.87	0.00
Interaction	2	0.03	0.97	0.00
*TR EMG amplitude*				
Working posture	1	6.82	**0.02**	0.18
Pointing device	2	1.69	0.20	0.02
Interaction	2	1.50	0.24	0.02
*DE EMG amplitude*				
Working posture	1	1.18	0.18	0.00
Pointing device	2	4.47	**0.02**	0.11
Interaction	2	0.18	0.84	0.02
*Task success rate*				
Working posture	1	0.39	0.54	0.00
Pointing device	2	14.56	**0.00**	0.32
Interaction	2	6.79	**0.00**	0.08
Note: Values in bold indicate practically significant effects.				
